# Afferents to Action: Cortical Proprioceptive Processing Assessed with Corticokinematic Coherence Specifically Relates to Gross Motor Skills

**DOI:** 10.1523/ENEURO.0384-23.2023

**Published:** 2024-01-19

**Authors:** Scott J. Mongold, Christian Georgiev, Thomas Legrand, Mathieu Bourguignon

**Affiliations:** ^1^Université libre de Bruxelles (ULB), UNI–ULB Neuroscience Institute, Laboratory of Neurophysiology and Movement Biomechanics, 1070 Brussels, Belgium; ^2^University College Dublin (UCD), School of Electrical and Electronic Engineering, D04 V1W8 Dublin, Ireland; ^3^Université libre de Bruxelles (ULB), UNI — ULB Neurosciences Institute, Laboratoire de Neuroanatomie et de Neuroimagerie translationnelles (LN2T), 1070 Brussels, Belgium; ^4^BCBL, Basque Center on Cognition, Brain and Language, 20009 San Sebastian, Spain

**Keywords:** EEG, gross motor skill, motor control, proprioception, sensorimotor cortex

## Abstract

Voluntary motor control is thought to be predicated on the ability to efficiently integrate and process somatosensory afferent information. However, current approaches in the field of motor control have not factored in objective markers of how the brain tracks incoming somatosensory information. Here, we asked whether motor performance relates to such markers obtained with an analysis of the coupling between peripheral kinematics and cortical oscillations during continuous movements, best known as corticokinematic coherence (CKC). Motor performance was evaluated by measuring both gross and fine motor skills using the Box and Blocks Test (BBT) and the Purdue Pegboard Test (PPT), respectively, and with a biomechanics measure of coordination. A total of 61 participants completed the BBT, while equipped with electroencephalography and electromyography, and the PPT. We evaluated CKC, from the signals collected during the BBT, as the coherence between movement rhythmicity and brain activity, and coordination as the cross-correlation between muscle activity. CKC at movements’ first harmonic was positively associated with BBT scores (*r* = 0.41, *p* = 0.001), and alone showed no relationship with PPT scores (*r* = 0.07, *p* = 0.60), but in synergy with BBT scores, participants with lower PPT scores had higher CKC than expected based on their BBT score. Coordination was not associated with motor performance or CKC (*p* > 0.05). These findings demonstrate that cortical somatosensory processing in the form of strengthened brain–peripheral coupling is specifically associated with better gross motor skills and thus may be considered as a valuable addition to classical tests of proprioception acuity.

## Significance Statement

Whether standing upright, jogging, or in Olympic competition, our nervous system not only sends out motor commands prompting muscles to contract but also receives incoming information to fine-tune motor actions. Though the machinery involved in sensing mechanical changes is well-described, the neural processing of this information is not, making its relevance to motor function unresolved. We found that the coupling strength between peripheral kinematics and cortical activity was related to motor function and at most, only weakly related to conventional muscle-only assessments. We present the novel behavioral relevance of this coupling and its specific relationship to gross motor skills. Our study paves the way for including novel brain-centered approaches to complement classical assessment sensorimotor functions in health and disease.

## Introduction

Proprioception is the sense of position and movement of our own body. While the “hardware” of proprioception, consisting of a collection of sensory mechanoreceptors located in muscles, tendons, joints, and the skin, provides kinematic data for the brain, central processing, akin to “software,” must integrate and use this information to update motor programs (Han et al., 2016; Imai and Yoshida, 2018; Palva and Palva, 2018; Tuthill and Azim, 2018). Currently, much of the literature describing the proprioceptive sense relies on peripheral proprioceptive acuity testing, with common tasks including threshold to detection of passive motion, joint position reproduction, or active movement extent discrimination. While these tasks have been useful in risk identification or in a variety of clinical and sport-specific settings (de Noronha et al., 2006; Hobbs et al., 2010; Mir et al., 2014), they do not describe the “software” of proprioception. Although the cortical and subcortical areas active during proprioceptive stimulation are well known (Goble et al., 2011), the neurophysiological mechanisms therein are still poorly described. The characterization of these mechanisms appears crucial considering that changes in movement sense are implicated across countless pathologies (Dietz, 2002), during aging (Ferlinc et al., 2019), and in competitive sports (Han et al., 2016).

It is clear that the field of motor control would benefit from an approach that examines how the brain tracks incoming proprioceptive information. A promising method in that respect is corticokinematic coherence (CKC), the coupling between cortical activity and limb kinetics or kinematics that occurs during repetitive movement tasks at movement frequencies (Bourguignon et al., 2011, 2012; Piitulainen et al., 2013a). Indeed, studies comparing CKC during active and passive movements and using directionality approaches suggest that CKC specifically assesses the cortical processing of somatosensory proprioceptive afferences (Piitulainen et al., 2013a, 2018a; Bourguignon et al., 2015). CKC studies have already revealed differences in proprioception in the case of children with cerebral palsy and elderly adults (Piitulainen et al., 2018b; Démas et al., 2022). Though CKC appears to be a viable tool to probe the neural tracking of proprioceptive information, its relationship to motor performance is yet unexplored.

Motor performance is a highly multidimensional concept. A common subdivision distinguishes between gross and fine motor skills. In this regard, the Box and Blocks Test (BBT) and the Purdue Pegboard Test (PPT) are regularly used in the rehabilitative medicine field to characterize these two aspects of motor performance. While both tasks require the use of the upper extremity, the BBT relies more on gross motor function, whereas the PPT necessitates more fine motor control (Gardner and Broman, 1979; Mathiowetz et al., 1985). Importantly, the highly repetitive nature of the BBT makes it an especially suitable task for CKC analysis (Marty et al., 2015a).

Yet another determinant contributor to motor performance is coordination (Bernshteĭn, 1967). In biomechanics, coordination is defined as the relation between muscle activations acting toward the achievement of a motor task (Kimura et al., 2021). The neuronal underpinning of coordination is not fully understood, but an increasingly acknowledged mechanism is the simultaneous control of multiple muscles by specialized spinal circuits, commonly known as muscle synergies (Saltiel et al., 2001; d’Avella et al., 2003; Kuppuswamy and Harris, 2014). Motor coordination can be assessed through the electromyographic (EMG) assessment of neuromuscular activations between muscles.

In this study, we estimated CKC between cortical activity and rectified EMG signals, as done previously (Piitulainen et al., 2013b; Bourguignon et al., 2019), with the aim of assessing its relationship to two types of motor skills, as measured by the BBT (gross) and PPT (fine). Additionally, coordination during the BBT was assessed to test its relationship with motor performance and with CKC. Correspondingly, we hypothesized that (1) CKC strength would be positively correlated with BBT and PPT scores and that (2) enhanced muscular coordination would coincide with better motor scores. Finally, since CKC is thought to represent the processing of incoming sensory afferents, based on previous directional coupling analyses (Piitulainen et al., 2013a; Bourguignon et al., 2015), and coordination to rely on spinal mechanisms for motor control, we hypothesized that (3) these two measures would be at most, weakly related.

## Methods

### Participants

A total of 61 healthy adults (32 females; mean ± SD age, 23.9 ± 3.5 years; range, 18–34 years) participated in the study. All participants were right-handed according to the Edinburgh Handedness Inventory (mean ± SD score, 72.6 ± 9.9; range, 57–90; Oldfield, 1971) and reported no history of neurological or motor impairments. Participants had no prior experience with the BBT and PPT. The protocol was approved by the ethics committee of the Université libre de Bruxelles. All subjects gave written informed consent in accordance with the Declaration of Helsinki.

### Experimental design

Participants were fitted with an EEG cap with 64 electrodes (waveguard original, ANT Neuro). Additionally, participants were fitted with electrodes on three muscles of the right upper extremity: first dorsal interosseous (FDI), biceps brachii, and anterior deltoid.

**Figure 1.**
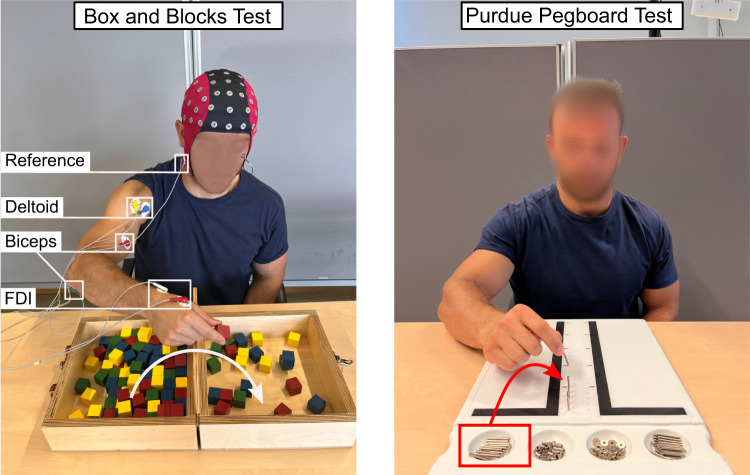
Experimental setup. Participants performed the BBT (left) while equipped with a 64-channel EEG cap and bipolar EMG electrodes on the FDI, biceps, and deltoid. Once a block is selected and picked up, the participant moves their hand over the barrier and releases the block. Participants performed the PPT (right) unequipped. Pegs were transferred from the starting position to the next available small hole.

Participants completed three trials of the BBT with the right hand (Fig. 1). The BBT requires a participant to transfer as many small wooden blocks as possible across a 15.2 cm tall barrier with the designated testing hand, during 1 min of testing (Mathiowetz et al., 1985). One hundred and fifty 2.5 cm^3^ wooden blocks were placed in random orientations in the partition on the side of the testing hand. A participant's score was equal to the number of blocks moved over the barrier, to an identical, but empty partition, in 1 min. The experimenter instructed the participants to move the blocks as quickly as possible and encouraged them throughout each trial.

Participants completed five trials of one subset of the PPT, in which they used only their right hand to transfer metal pegs from their starting position, in the upper right-hand side of the board, to the right column with small holes spaced along the length of the board (Fig. 1). Each participant was instructed to transfer as many pegs as possible in 30 s.

The experimenter performed one trial of the BBT and PPT to show the participant how to perform it properly.

### Data acquisition

EEG and EMG signals were recorded during the BBT trials. The EEG signals were continuously recorded with a conventional cap in a layout based on the extended international 10–20 system for electrode placement. The nasion, inion, and midsagittal plane of the head were used as anatomical landmarks to place the cap. The EEG cap was connected to an EEG mobile amplifier (eegoTM, ANT Neuro). Electrode-skin impedance levels were kept below 20 kΩ with electrolyte gel. Brain activity was visualized and sampled at 1,000 Hz using the eegoTM software (v1.9.1; ANT Neuro) running on an interactive tablet.

The EMG signals were continuously recorded with bipolar electrodes. EMG electrodes were connected directly to the EEG amplifier and signals were recorded on auxiliary channels of the EEG amplifier. EMG signals were sampled at 1,000 Hz. Proper placement of EMG electrodes on respective muscle bellies was verified during isometric contractions.

### Data preprocessing

EEG signals were imported into MATLAB (R2019b, MathWorks) where custom scripts combined with functions from the FieldTrip toolbox (Donders Institute for Brain, Cognition, and Behavior; Oostenveld et al., 2011) were used for data analysis. EEG signals at electrodes affected by excessive noise levels (mainly due to bad electrode-skin contact) were reconstructed by interpolation of the signals from the surrounding electrodes as done previously (Perrin et al., 1989). Electrodes were considered noisy when they matched at least one of the three criteria: (1) too high wide-band amplitude, (2) too high ratio between high- and low-frequency amplitudes, and (3) too low correlation with other channels, as proposed previously (Bigdely-Shamlo et al., 2015). Details of the methods and default values used for these three criteria can be found in Bigdely-Shamlo et al. (2015). EEG signals were then re-referenced to a common average. Thereafter, independent component analysis was applied to the EEG signals filtered through 0.5–45 Hz, with the EEG data being decomposed into 20 independent components using the FastICA algorithm (dimension reduction, 25; nonlinearity, tanh; Hyvärinen et al., 2000; Vigario et al., 2000). The spatial distribution and time series of each component were visually analyzed for each trial, with 1–6 components corresponding to eyeblink and heartbeat artifacts, or nonuniform noise selected for removal. The corresponding components were subsequently subtracted from raw EEG signals.

**Figure 2.**
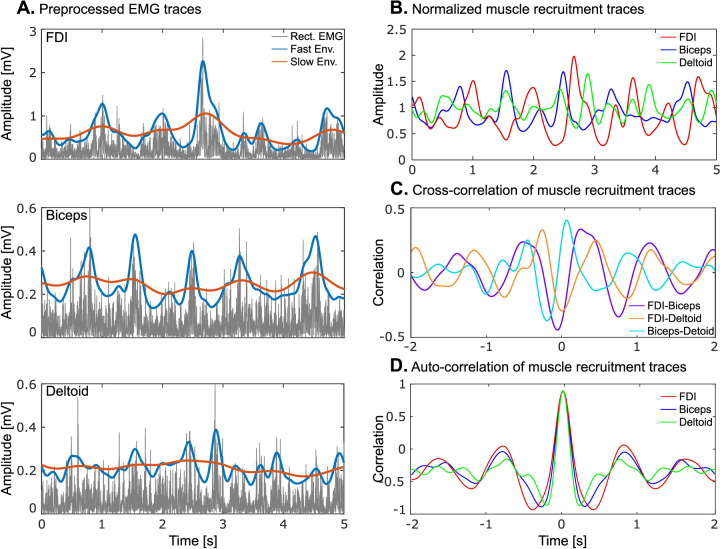
Processing of EMG signals. ***A***, Excerpt of pre-processed EMG signals for the FDI, biceps, and deltoid from a representative participant. From each of the rectified EMG signals (dark gray traces) were extracted a fast (blue traces) and a slow (red traces) envelope. ***B***, Muscle recruitment traces for each of the muscles, obtained as the ratio between the fast and slow envelopes. ***C***, Cross-correlations between pairs of muscle recruitment traces. Their maximal amplitude was taken as an estimate of motor coordination. ***D***, Auto correlations for the same muscle recruitment traces. The amplitude of their side peaks was taken as a measure of regularity.

As a preliminary step to correct for slow drift, movement, and power-line artifacts, EMG signals recorded during the BBT trials were filtered between 20 and 295 Hz with notch filters at 50 Hz and harmonics using a zero-lag fast Fourier transform-based filter and rectified (Fig. 2*A*). We then applied a processing procedure to correct for changes in EMG amplitude across cycles. To do this, fast and slow envelopes were extracted from each rectified EMG by smoothing it with a Gaussian kernel that implements a low-pass filter at 3 and 0.7 Hz, respectively (Fig. 2*A*). These frequencies were chosen to capture the range of expected movement frequencies (1–1.5 Hz). The ratio of the fast envelope and the slow envelope represents the muscle recruitment trace (Fig. 2*B*). It emphasizes the periodicity of the individual muscle activity within each movement cycle while removing the influence of nonstationarity across cycles.

### EMG processing

During the BBT, three parameters of muscle activity were extracted from the upper extremity: coordination, regularity, and EMG modulation depth.

Motor coordination was assessed with two different methods: cross-correlation and mutual information. Both are fit for clinical translation as neither is computationally demanding. Cross-correlation is an established method for comparing EMG signals between different muscles (Hawkes et al., 2012). This approach measures the similarity in shape between two curves, with regard to the temporal characteristics of the signals (Hawkes et al., 2019). Here, the Pearson correlation coefficient was estimated between each pair of the three muscle recruitment traces with delays ranging from −2 to 2 s, by steps of 20 ms (Fig. 2*C*). For each pair, the maximum in absolute value was retained, and the average across pairs was taken as the correlation-based coordination measure. Mutual information analysis has been established as another similarity index, with successful use in assessing motor coordination (Samani et al., 2017). Importantly, this method detects linear and nonlinear statistical dependencies between time series, whereas cross-correlation is limited to the assessment of linear dependence (Jeong et al., 2001). Upon analysis of the activity of the deltoid muscle across participants, we noticed a biphasic activation pattern along the movement cycle. Therefore, the mutual information approach was adopted to capture potential nonlinear relationships between signals that otherwise would not have been identified. Mutual information was estimated between each pair of the three upper extremity muscle recruitment traces. Three types of mutual information were computed and averaged across muscle pairs: (1) mutual information directly applied to the muscle recruitment traces; (2) mutual information applied to the phase of the muscle recruitment traces, as extracted with the Hilbert transformation; and (3) permutation mutual information (Cheng et al., 2018). In the two first cases (1 and 2), mutual information was applied to the data quantized into 10 equally populated bins. In the last case (3), mutual information was applied to the permutation order derived from four adjacent time points with a distance of 100 ms. Thus, permutation mutual information determined whether the order obtained by sorting the values over the four time points in one signal tended to predict the presence of one or more other orders in the other signal.

Regularity was assessed because it could bias our estimation of coordination and CKC, the latter two being expected to increase with more regular movements. Regularity can be defined as the consistency of a given muscle activation pattern, where high regularity corresponds to similar activation patterns across cycles of a given task and low regularity results from heterogeneous activation patterns. With this view in mind, regularity can be assessed using autocorrelation, as done more commonly in gait kinematic investigations (Kobayashi et al., 2014) and in studies of sensorimotor coordination (Carroll et al., 2001), where the EMG signal for a designated muscle is compared to itself across successive time lags. The autocorrelation coefficient is 1 at time 0 and decreases with increasing delays imposed on the signal. However, in rhythmic activities, positive side peaks appear at integer multiples of cycle duration with negative side peaks in between; the more regular the signal, the greater the magnitude of the side peaks. Therefore, we estimated the Pearson autocorrelation for three muscle recruitment traces with delays ranging from −2 to 2 s, by steps of 20 ms, and quantified regularity with the magnitude in the absolute value of the first negative and positive side peaks (Fig. 2*D*).

The modulation depth of muscle recruitment traces was assessed to quantify the salience of rhythmic EMG activity. It was quantified with the coefficient of variation, later averaged across the three muscles. This parameter is important to control for as it has the potential to bias regularity, coordination, and CKC measures. Indeed, the latter three measures are expected to decrease with decreasing modulation depth, simply because movement cycles become less clearly represented.

### EEG processing

CKC was calculated for the three 1-min-long trials of the BBT combined. The coherence was computed between rectified FDI muscle activity and each EEG signal. Noteworthily, a rectified EMG signal picks up movement rhythmicity much like an accelerometer, so that its coherence with EEG signals at movement frequencies can be safely termed CKC (Piitulainen et al., 2013b; Bourguignon et al., 2019). Coherence is an extension of the Pearson correlation coefficient to the frequency domain, which quantifies the degree of coupling between two signals, for example, the CKC strength, by providing a number between 0 (no linear dependency) and 1 (perfect linear dependency) for each frequency (Halliday et al., 1995).

For coherence analyses, the continuous data were split into 5 s epochs with 4 s epoch overlap, leading to a frequency resolution of 0.2 Hz (Bortel and Sovka, 2007). EEG epochs where the signal amplitude of at least one electrode exceeded five standard deviations above the mean were excluded to avoid contamination of the data by internal or external noise sources. We then performed a coherence analysis (Halliday et al., 1995), yielding cross-, power, and coherence spectra—between EEG and rectified EMG signals. The magnitude squared coherence was chosen as the coupling measure as done in previous CKC studies (Bourguignon et al., 2011, 2012, 2013, 2015; Piitulainen et al., 2013b, 2015; Marty et al., 2015b). Individual power and coherence spectra were used to identify peak CKC, which occurred at the approximate movement frequency (F0) and its first harmonic (F1). Maximum values at F0 and F1 across all electrodes, excluding those located on the edge of the cap, were extracted for each individual. Topographic distributions of CKC, visualized using the FieldTrip toolbox (Halliday et al., 1995; Oostenveld et al., 2011), confirmed that peaks in coherence were identified in the neighborhood of electrodes above the primary sensorimotor cortex (SM1), contralateral to the movement.

The SNR of the EEG signal is a potential confounding factor in coherence analysis, where decreased SNR leads to decreased coherence. To take this factor into account, we assessed it at F0 and F1, for the electrode with maximum CKC. It was estimated as the power at the considered frequency (F0 or F1) divided by the geometric mean of the power ±2 frequency bins away.

### Code accessibility

The code/software described in the paper is freely available online at https://osf.io/46bsg/?view_only=a6355d5112bd4e878525814ed8ca6b9e. The code is available as Extended Data.

10.1523/ENEURO.0384-23.2023.s1-1 Extended Data Download Extended Data, ZIP file.

### Statistical analysis

Individual CKC values were compared against a surrogate distribution to determine significance. Surrogate coherence was evaluated between original EEG and Fourier transform surrogate reference signals (1,000 repetitions). The Fourier transform surrogate of a signal is obtained by computing its Fourier transform, replacing the phase of the Fourier coefficients by random numbers in the range (−π; π) and then computing the inverse Fourier transform. The threshold for statistical significance was set to *p* < 0.05 and was obtained as the 95th percentile of the distribution of the maximum coherence across 1–4 Hz and across all considered electrodes.

Correlation between motor performance scores (BBT and PPT), EMG-based parameters, and CKC values were assessed via Spearman's rank-order correlation test. It should be noted that a very strong correlation (*r* > 0.8) was identified within the four measures of motor coordination and within the two measures of regularity. Therefore, related parameters were combined into a unique measure by averaging them after classical standard normalization (mean subtraction and division by the standard deviation).

Potential confounding factors for regularity, coordination, and CKC (our dependent variables) were assessed by correlation and corrected when the correlation was statistically significant. Specifically, when a significant correlation was identified with a confounding variable, the mean subtracted confounding variable was regressed out of the dependent variable, resulting in a new corrected dependent variable. When more than one confounding factor was identified, they were regressed out sequentially, taking care of orthogonalizing each of them with respect to the previously removed ones. For regularity, the potential confounding factor was modulation depth as clearer variations in the EMG envelope may correspond to increased regularity. For coordination, potential confounding factors were regularity and modulation depth. For CKC, potential confounding factors were modulation depth, regularity, and EEG SNR.

A partial information decomposition (PID; Ince, 2017; Ince et al., 2017) analysis was used to dissect the information about CKC brought by motor performance. PID is a method to describe the extent by which two explanatory variables bring unique information about a target (information present in one variable but not in the other), redundant information (information common to the two variables), and synergistic information (information emerging from the interaction of the two variables; Kunert-Graf et al., 2020). In this way, PID was used to estimate the unique, redundant, and synergistic information brought by BBT and PPT performance about CKC. PID analysis was implemented in MATLAB, from all three variables with Gaussian copula rank normalization done (Williams and Beer, 2010; Ince et al., 2017; Park et al., 2018). Following, partial information was calculated with a redundancy lattice as done previously (Williams and Beer, 2010; Ince et al., 2017; Park et al., 2018). For statistical assessment and conversion into easily interpretable *z* scores, measures of information were compared to the distribution of these measures obtained after permuting motor scores across subjects (1,000 permutations).

## Results

### Gross and fine motor skill assessment

Participants’ scores on the BBT, which assesses gross motor skills, ranged from 54 to 98 blocks transported during a single 1 min trial, with an average (±SD) of 76.2 (±7.6) blocks. These scores are compatible with existing normative data of healthy adults, as reported in females, 78 (±10.4), and males, 77 (±11.6; Mathiowetz et al., 1985).

On the more dextrous task, the PPT, participants’ scores ranged from 11 to 19 inserted pegs, with an average of 15.7 (±1.7) pegs inserted. These scores are comparable to normative data from young, healthy adults as reported in females, 16.6 (±2.1), and males, 15.7 (±1.7; Yeudall et al., 1986).

**Figure 3.**
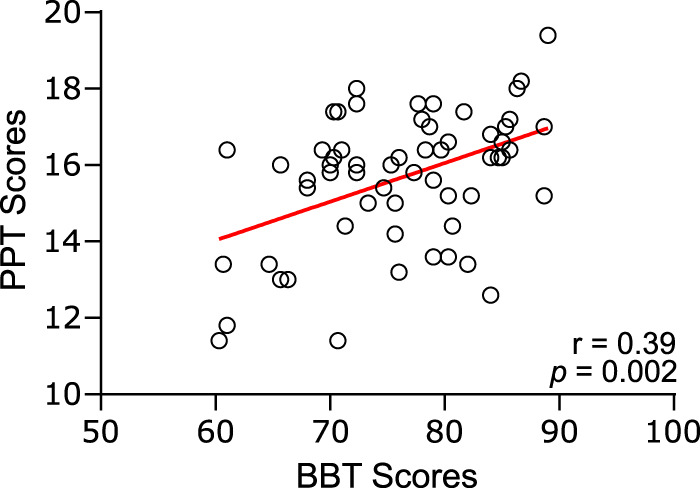
Relationship between PPT scores (pegs inserted in 30 s) and BBT scores (blocks transferred in 1 min). Circles indicate individual values, and their linear regression line is in red. The correlation value and associated significance level are indicated in the bottom right corner.

Figure 3 presents the relation between BBT and PPT scores. Both were significantly correlated (*r* = 0.39, *p* = 0.002). However, the magnitude of this association was not particularly strong, confirming that BBT and PPT assess partially different aspects of motor performance. Overall, existing variability and decoupling in test scores lend support to the notion that participants possess differing motor control profiles.

### EMG modulation depth, regularity, and coordination

The EMG modulation depth across participants possessed a mean (0.23) that was relatively high compared to its SD (0.03), indicating that the modulation of muscle activity was captured similarly across participants.

The two measures of regularity, including the negative and positive autocorrelation side peaks, were highly related (*r* = 0.81, *p* < 0.0001). Therefore, these two measures were combined into one measure of regularity of muscle activity. This resulting measure of regularity showed a significant positive correlation with EMG modulation depth (*r* = 0.49, *p* < 0.0001). To account for this association, the regularity measure was corrected for EMG modulation depth.

The four coordination measures extracted from the cross-correlation and mutual information analyses were highly related (0.80 < *r* < 0.95, *p* < 0.0001). Therefore, these measures were combined into a unique measure of coordination. The resulting coordination measure was significantly correlated with EMG modulation depth (*r* = 0.59, *p* < 0.0001). Consequently, the coordination measure was corrected for EMG modulation depth. In addition, we identified another significant relationship between corrected coordination and corrected regularity measures (*r* = 0.76, *p* < 0.001). Therefore, the coordination measure was corrected for both EMG modulation depth and regularity.

Notably, there was no significant correlation between regularity and BBT (*r* = 0.15, *p* = 0.24) or PPT scores (*r* = 0.12, *p* = 0.37) or between coordination and BBT (*r* = −0.01, *p* = 0.92) or PPT scores (*r* = 0.07, *p* = 0.62).

### CKC

**Figure 4.**
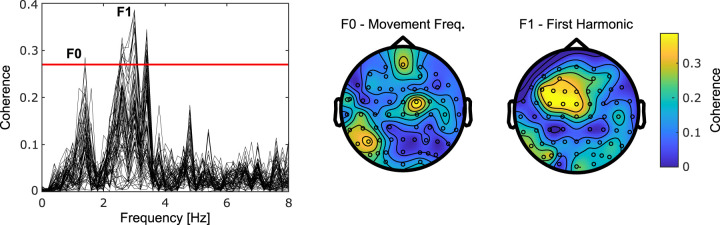
Spectrum and scalp distribution of CKC for a representative individual. The CKC spectrum presents one trace for each EEG electrode, and a horizontal red line indicates the level of statistical significance. Coherence peaked at F0 and F1, corresponding to ∼1.5 and 3 Hz, respectively, for this particular individual. Scalp distributions are mostly compatible with tangential sources in the left SM1 cortex, although other sources might have contributed (Bourguignon et al., 2012).

Figure 4 presents a characteristic coherence spectrum and topography found for the coupling between cortical activity and rectified FDI muscle activity during the BBT. In line with previous studies (Bourguignon et al., 2011, 2012; Piitulainen et al., 2013a, 2018a, 2020; Marty et al., 2015a), such CKC peaked at F0 and F1. Also, the topography at each frequency showed a tendency for CKC to peak approximately around the C3 electrode, compatible with SM1 activation, with sources at times radially or tangentially oriented. Overall, 31 participants showed significant CKC at F0, representing 52% of our sample, while 41 participants had significant CKC at F1, accounting for 67% of our sample. Interestingly, it was not uncommon for CKC to be higher at F1 (0.23 ± 0.10) compared to F0 (0.20 ± 0.11), but this difference was not significant (*p* = 0.08; Wilcoxon test).

### Behavioral relevance of CKC

**Figure 5.**
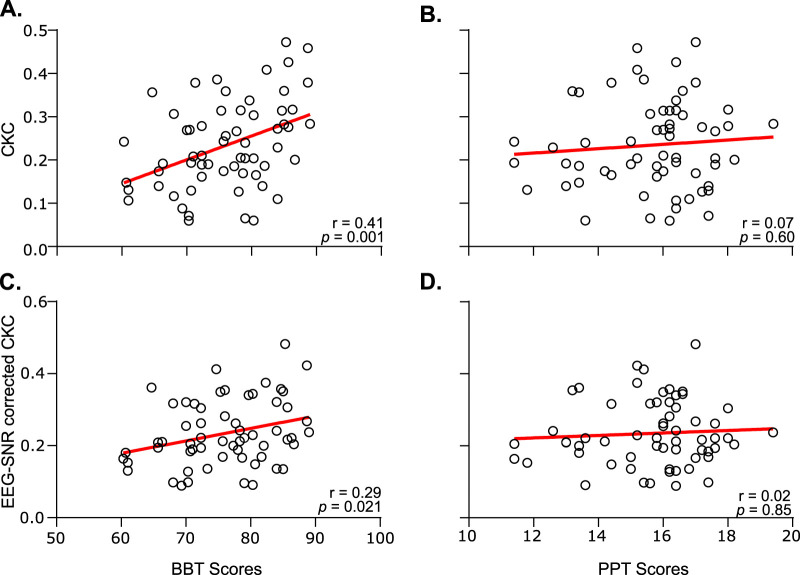
Relation of CKC at F1 with BBT scores (***A***), PPT scores (***B***), and EEG SNR corrected CKC at F1 with BBT scores (***C***) and PPT scores (***D***). Plots are as in Figure 3.

Correlations of CKC at F0 and F1 with the BBT and PPT scores yielded different results. At F0, CKC was not significantly associated with BBT scores (*r* = −0.02, *p* = 0.88) or PPT scores (*r* = −0.11, *p* = 0.40). However, at F1, CKC was significantly associated with BBT scores (*r* = 0.41, *p* = 0.001; Fig. 5*A*) but not with PPT scores (*r* = 0.07, *p* = 0.60; Fig. 5*B*).

Additionally, we investigated whether the association between CKC at F1 and performance could be explained by confounding factors. As a result, CKC was not significantly associated with EMG modulation depth (*r* = 0.002, *p* = 0.99) or regularity (*r* = −0.009, *p* = 0.94). These results indicate that the EMG SNR was high enough in all individuals not to noticeably influence CKC values and that the range of movement regularity was restricted enough not to smear CKC peaks in some individuals more than in others. Our last step to deal with potential confounding factors involved the inclusion of the SNR from EEG activity. CKC at F1 was significantly associated with the SNR (*r* = 0.48, *p* < 0.0002) and hence corrected for it. After correction, CKC at F1 was still significantly associated with BBT scores (*r* = 0.29, *p* = 0.021; Fig. 5*C*). Still, after correction, PPT scores were not significantly associated with CKC at F1 (*r* = 0.02, *p* = 0.85; Fig. 5*D*).

Finally, we tested our hypothesis that CKC is at most weakly related to motor coordination. Our data was in line with this hypothesis, revealing nonsignificant correlation between coordination and CKC at F0 (*r* = 0.24, *p* = 0.07) and F1 (*r* = 0.16, *p* = 0.24). And when CKC data was corrected for EEG SNR, there was a significant correlation at F0 (*r* = 0.29, *p* = 0.02), but not at F1 (*r* = 0.20, *p* = 0.12).

### PID

**Figure 6.**
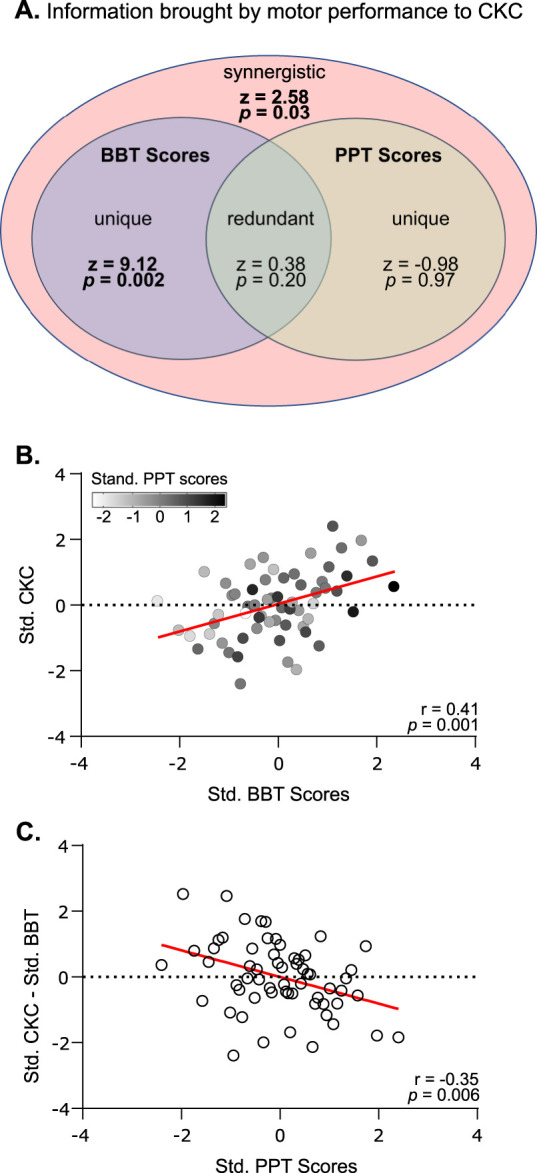
Fine-grained relationship of CKC with motor skills. ***A***, Information structure of BBT and PPT scores predicting CKC. Ellipses outline the decomposition of mutual information into unique information brought by BBT scores (purple), unique information brought by PPT scores (beige), redundant information present in both scores, and synergistic information not present in either score but emerging from them together. Their significance level is indicated in each partition. ***B***, Relationship between standardized CKC and standardized BBT scores, where individual values are color-coded for their standardized PPT scores. ***C***, Relationship between standardized BBT scores subtracted from standardized CKC as a function of PPT scores. Plots are as in Figure 3.

Figure 6*A* represents the information structure of BBT scores and PPT scores as related to the prediction of CKC. BBT scores brought significant unique information about CKC (*z* = 9.12; *p* = 0.002), whereas PPT scores did not (*z* = −0.98; *p* = 0.97). Together, the two scores did not bring significant redundant information (*z* = 0.38; *p* = 0.20), but their simultaneous observation did bring synergistic information about CKC (*z* = 2.58; *p* = 0.03).

Figure 6*B* characterizes how CKC varies according to BBT and PPT scores. Clearly, from the correlation analysis, CKC is best predicted by BBT scores compared to PPT scores, and this is supported by the difference in unique information that each brings to CKC. However, when plotting the variables together (Fig. 6*B*), it becomes apparent that the knowledge of the PPT scores could help refine CKC prediction beyond its regression line on BBT scores. Participants with high PPT scores tended to display reduced CKC compared to what is expected from their BBT score (below the regression line). The same conclusions can be drawn when standardized BBT scores are subtracted from standardized CKC, and this contrast is plotted against PPT scores (Fig. 6*C*). Indeed, when PPT scores are low, the contrast was high, meaning that a participant had a CKC value that was higher than expected from their BBT score, and vice versa, as attested by a significant negative correlation (*r* = −0.35, *p* = 0.006).

## Discussion

In this study, we examined the relationship between CKC, a marker of cortical proprioceptive processing, and motor performance, with the aim of establishing the behavioral relevance of CKC. We identified a significant relationship between CKC at F1, but not F0, and motor performance of a gross motor control task. Conversely, the motor performance of a fine motor control task was informative about CKC, but only when considered in synergy with gross motor ability. Finally, motor coordination did not contribute to motor performance but showed a trend of association with CKC at F0, but not F1.

### Behavioral relevance of CKC

In our study, higher BBT scores, indicating more favorable gross motor function, corresponded to stronger CKC. This finding suggests that there is a behavioral advantage to stronger CKC on this motor task. In the following paragraphs, we present several nonexclusive explanations for this behavioral advantage.

Previous analyses have shown that CKC likely reflects the processing of afferent somatosensory, mostly proprioceptive, information (Piitulainen et al., 2013a; Bourguignon et al., 2015). Therefore, increased CKC could indicate an increased quantity of cortical resources allocated for sensory processing. Following the views whereby inefficient neural coding could be compensated by recruiting additional neural populations (Nurmi et al., 2021), stronger CKC may represent an increase in the utilization of neural substrate. This idea has been brought forward in another study in which stronger CKC was associated with worse postural stability (Piitulainen et al., 2018b). But unlike during postural balance, where the consistent need for more neural resources may be computationally unfavorable, the ability to allocate additional neural substrates for a novel task, like the BBT in our study, could foster better integration of afferent signals. Accordingly, the interpretation of CKC magnitude appears to be task dependent.

The relationship between CKC and task performance might also be explained by a more robust cortical representation of proprioceptive afferences in the best performers. This is well in line with the behavioral relevance of neural entrainment to external stimuli seen across a range of modalities, best explored in language literature where entrainment to speech benefits comprehension (Ahissar et al., 2001; Peelle et al., 2013; Vanthornhout et al., 2018; Bertels et al., 2023), as well as in the context of sign language observation (Malaia et al., 2021). Alternatively, higher BBT scores are likely to be achieved with faster movements. Since muscle spindle activity has been shown to increase with faster movement (Sasaki et al., 2018), it should result in stronger afferent volleys to the SM1 and hence increased CKC. Attention and motivation may also vary across participants and impact both performance and CKC through the neuronal resources allocated to the task. Thus, changes in CKC, similar to corticomuscular coherence, could be related to attention-based parameters (Kristeva-Feige et al., 2002; Johnson et al., 2011).

Beyond the behavioral relevance of CKC for gross motor function, our PID analysis revealed that fine motor control, in the form of PPT scores, brought more intricate synergistic information about CKC when observed together with BBT scores. We show that individuals with enhanced fine motor skills (high PPT) tend to possess lower levels of CKC than expected, based on their gross motor skills (BBT), with the converse also holding true. This suggests that CKC is higher in relation to features present in the BBT, but not in PPT. While both tasks rely on gross control of the arm and forearm, the performance of the BBT likely depends on accurate proprioceptive encoding of upper limb joint positions and movements, while the PPT may rely more on visuomotor integration as required by the precise placement of the peg (Hinkle and Pontone, 2021), giving less weight to proprioception. These views lay strong support to the notion that CKC is mainly driven by proprioceptive afferences (Piitulainen et al., 2013a; Bourguignon et al., 2015), while adding the important notion of behavioral relevance.

In assessing the relationship between CKC and BBT values, we identified the need to correct CKC values for EEG SNR, leading to a slight decrease in correlation strength from 0.41 without correction to 0.29 with correction. However, this drop does not necessarily indicate that the initial association between noncorrected CKC values and BBT scores was inflated. Indeed, correcting CKC values for EEG SNR was meant to control for a bias, but also removed part of the effect we meant to identify. Regarding the bias, it could arise because the EEG noise level typically decreases with the frequency (Demanuele et al., 2007), leading to higher CKC values at higher frequencies if the amplitude of the genuine cortical signal is constant. However, a previous study, though conducted on a smaller sample size than the present one, reported that CKC was not affected by movement frequency within the range of 1–3 Hz (Marty et al., 2015b). Regarding the second point, our hypothesis was that of an association between CKC and motor performance, which in the case of BBT is proportional to movement frequency. Yet, the coherence is well known to increase with genuine signal amplitude, along with better phase locking. Hence, under our hypothesis, it was expected that EEG SNR would increase with motor performance. Therefore, the correlation of 0.29 obtained with corrected CKC values should be taken as a lower bound for the strength of the association between CKC and motor performance.

While we cannot argue that motor performance depends on genetic factors giving rise to increased or decreased sensory processing ability or occurs as a result of a lifetime of motor practice, there is evidence for both to be true. Recently, the *Piezo2* gene, expressed primarily in peripheral sensory neurons, but also found in the brain, has been linked to interindividual variability in the functionality of mechanosensation (Nagel and Chesler, 2022), suggesting the efficacy of proprioceptive processing could be dependent on genetics. Alternatively, motor skill practice and motor learning are two extremely well-described behaviors known to modify brain activity (Kantak and Winstein, 2012).

### Coordination

In using EMG recordings during the BBT, we assessed muscle coordination and identified how it relates to motor performance and CKC. Here, coordination refers to the similarity in muscle activation patterns across movement cycles.

We did not identify a significant relationship between muscle coordination and BBT scores. Although we expected that increased coordination values would coincide with higher BBT scores, our result is not entirely surprising. In the BBT, blocks sit randomly within one partition. Participants must adjust each block transfer depending on their selection, resulting in variable joint trajectories (Hebert et al., 2014) and correspondingly variable muscle activation patterns. Therefore, a flexible motor control strategy in which muscle activation patterns differ depending on block selection may point towards efficient motor control, while coordination, as defined in this study, is less relevant.

Correlations for CKC at F0 and F1 and coordination revealed no significant relationships, except for a weak one when CKC at F0 was corrected for EEG SNR. These findings support our initial hypothesis that CKC and coordination hinge on different mechanisms.

### Limitations and perspectives

Owing to the dynamic nature of the task, our EEG recordings were contaminated to some degree with movement artifacts. During the BBT, participants were instructed to keep their heads still and facial muscles relaxed, but in practice, this was challenging. This likely reduced the precision of CKC estimation and could explain the lack of relationship identified between motor performance and CKC at F0. Artifacts were noticeable as increased coherence at electrodes on the edge of topographic plots, which is why these were excluded from the analyses.

Another reason why CKC at F0 was not found to be behaviorally relevant could rest in its susceptibility to heartbeats and resultant artifacts, which tend to occur around the same frequency. However, heartbeat artifacts were sought and suppressed with independent component analysis. Alternatively, previous studies have also suggested that CKC at F1 may not merely represent the harmonic of CKC at F0 (Pollok et al., 2004, 2005; Bourguignon et al., 2012; Piitulainen et al., 2013a). Simple flexion/extension movements could arise due to the integration of the two volleys of sensory information caused by flexion and the extension within a movement cycle (Pollok et al., 2004, 2005; Bourguignon et al., 2012; Piitulainen et al., 2013a). Likewise, during the BBT, participants performed back-and-forth movements during one cycle.

Critically, the main findings of this study are correlative in nature, meaning they do not disentangle causes and consequences. Future studies designed to modulate cortical processing of somatosensory information (e.g., via stimulation) or motor dexterity (e.g., via training) may clarify the mechanisms underlying the identified relationships.

Still, the present study opens up important perspectives for future clinical studies and practice. That is, CKC and coordination represent informative measures that could be gathered in the framework of the BBT, providing a more global view of a patient's motor control and hopefully improving clinical decision-making.

Lastly, it could be argued that CKC should be interpreted only in the context of the task from which it is derived—BBT in our case. Nevertheless, previous work has established that CKC measured in one context could be related to performance in another completely different task. Specifically, CKC measured during passive ankle movements was found to be related to the amplitude of standing postural sway (Piitulainen et al., 2018b). Hence, CKC may represent a global index of proprioceptive processing, suggesting it is warranted to compare CKC across task types. Therefore, in aiming to develop a nuanced understanding of CKC's relationship to gross and fine motor control, we compared CKC magnitude to BBT and PPT performance. Future work investigating how CKC varies across tasks in the same individual should clarify to which extent CKC reflects some innate cortical proprioceptive processing ability as opposed to task-specific demands.

### Conclusion

Our study confirms the ability to evaluate CKC during a dynamic and variable task such as the BBT and establishes CKC as a behaviorally relevant marker of effective cortical proprioceptive processing, where high values of CKC are predictive of good gross motor function. CKC measurement may favorably complement classical behavioral assessments of proprioception in contexts where proprioceptive acuity is in question, help discern if the brain is involved in proprioceptive deficits, or assess plasticity related to improving cortical processing.

## Synthesis

Reviewing Editor: Jennifer Dulin, Texas A&M University Decisions are customarily a result of the Reviewing Editor and the peer reviewers coming together and discussing their recommendations until a consensus is reached. When revisions are invited, a fact-based synthesis statement explaining their decision and outlining what is needed to prepare a revision will be listed below. The following reviewer(s) agreed to reveal their identity: Markus Butz.

The manuscript describes the relationship between motor performance and corticokinematic coherence, based on a large EEG study. Both reviewers agreed that the manuscript highlights novel findings that would be of interest to the broader neuroscience community, but there are some issues with the paper that preclude publication in eNeuro. First, the authors should clarify the rationale for correlating CKC values with the PPT task. Both reviewers agreed that there is a need for more detailed description of the statistical analysis methods used.

Reviewer #1:

In their manuscript „Afferents to Action: Cortical proprioceptive processing assessed with corticokinematic coherence specifically predicts gross motor skills" the authors study the link between motor performance and corticokinematic coherence (CKC). CKC was found to be correlated with gross motor skills (Box and Blocks Test), but not with fine motor skills (Purdue Pegboard Test) in case fine motor skills were regarded alone.

The research question under study is relevant. The study is well designed and uses well established methods in a straight forward way. The chosen tests are specifically well established for clinical populations and widely used, which increases the relevance of the findings. Moreover, it is positive that results are based on a relatively high number of participants (n=61) for a EEG study. The results are convincing, but the novelty is limited. However, I would consider the data at hand as a useful minor addition to the state-of-the-art. All in all, this is a well-written manuscript which is concise and solid as it is.

There are some points which might benefit from attention by the authors:

(i) In the title, the authors use the word 'predict'. Is this really justified, as the results are based on correlations which does not imply causality? The aspect of correlations as main findings might also be covered in the discussion e.g. in the limitation section.

(ii) Why do the authors use the term 'corticokinematic coherence', although they use the EMG as a reference? I was expecting a kinematic recording from the title such as an accelerometer. Would corticomuscular coherence not be more intuitive here? Moreover, do we really know how much of CKC is really proprioception and how much is motor command? And how far can we disentangle both?

(iii) Already in the abstract the authors might want to report statistical results for their correlation analyses. This would be useful for the readers.

(iv) The term 'motricity' in the discussion might not be familiar to all readers and might benefit from some explanation.

(v) I'm not sure that faithful/faithfully are the most fitting words as used here.

(vi) Please use 's' for seconds throughout the entire manuscript including legends instead of spelling it out.

(vii) The citations Bernshtein (1967) and Hyvärinen et al (2001) are incomplete.

Reviewer #2:

In this study, the authors carried out EMG and EEG recordings in human subjects performing a gross motor task ("BBT"), and separately had the same subjects perform a fine motor task ("PPT"). In addition to the motor performance scores themselves, they carry out a number of rigorous quantifications based on the EEG and EMG data acquired during the BBT task. These include: (i) muscle coordination - a measure of how well the activation pattern of each muscle predicted that of others, (ii) regularity - a measure of the periodicity of each muscle activation, and (iii) "CKC" - the coherence between the EMG and EEG data as a function of frequency. Their main finding is that high CKC values at the first harmonic of the movement frequency in the primary sensorimotor area was associated with better performance in the BBT task (Figure 4 and Figure 5A), which is an interesting result and should be reported.

However, the data for the PPT task and its relationship with the rest of the data are, in my opinion, much harder to interpret since they represent an independently acquired dataset (albeit from the same subjects). The authors find that CKC values have no relationship with PPT performance (Figure 5B). I am not sure why we should expect one in the first place, since the PPT is an entirely different task and BBT and PPT scores were only weakly correlated with each other. The authors also find that subjects with high PPT scores tended to have lower CKC values than one would expect given their BBT scores - but this is also difficult for me to interpret since I lack a clear intuition for how the measured CKC values might relate (if at all) to the PPT task.

Minor:

In the Results section, the authors report that several measures were correlated with each other and that this was somehow corrected for (for instance, correlation between coordination measure and EMG modulation depth). It is unclear to me how this correction was carried out. It might also be helpful to directly show the result of the correction by plotting the correlated measures against each before and after the corrections.

## Author Response

Response to Reviews - eNeuro

Manuscript title: Afferents to Action: Cortical proprioceptive processing assessed with corticokinematic coherence specifically relates to gross motor skills

Synthesis of Reviews:

Synthesis Statement for Author (Required):

The manuscript describes the relationship between motor performance and corticokinematic coherence, based on a large EEG study. Both reviewers agreed that the manuscript highlights novel findings that would be of interest to the broader neuroscience community, but there are some issues with the paper that preclude publication in eNeuro. First, the authors should clarify the rationale for correlating CKC values with the PPT task. Both reviewers agreed that there is a need for more detailed description of the statistical analysis methods used.

Response to the Synthesis Statement:

We thank both reviewers and the editor for their valuable feedback. For the rationale for correlating CKC values with the PPT task, we explicitly describe this reasoning in a comment from Reviewer #2. Ultimately, we feel justified in including PPT comparisons, but leave its inclusion up to the reviewers. In regards to the second comment, we have addressed the relevant statistical methods where applicable.

-

Reviewer #1:

In their manuscript "Afferents to Action: Cortical proprioceptive processing assessed with corticokinematic coherence specifically predicts gross motor skills" the authors study the link between motor performance and corticokinematic coherence (CKC). CKC was found to be correlated with gross motor skills (Box and Blocks Test), but not with fine motor skills (Purdue Pegboard Test) in case fine motor skills were regarded alone.

The research question under study is relevant. The study is well designed and uses well established methods in a straight forward way. The chosen tests are specifically well established for clinical populations and widely used, which increases the relevance of the findings. Moreover, it is positive that results are based on a relatively high number of participants (n=61) for a EEG study. The results are convincing, but the novelty is limited. However, I would consider the data at hand as a useful minor addition to the state-of-the-art. All in all, this is a well-written manuscript which is concise and solid as it is.

There are some points which might benefit from attention by the authors:

(i) In the title, the authors use the word 'predict'. Is this really justified, as the results are based on correlations which does not imply causality? The aspect of correlations as main findings might also be covered in the discussion e.g. in the limitation section.

Response to (i): We agree with the reviewer that our experiment was not designed to test causality. Therefore, we now use "relates to" in place of "predicts".

Correlations as main findings are now included in the discussion (limitation) section (see lines 492-495):

"Critically, the main findings of this study are correlative in nature, meaning they do not disentangle causes and consequences. Future studies aiming to modulate cortical processing of somatosensory information (e.g. via stimulation) or motor dexterity (e.g., via training) may clarify the mechanisms underlying the identified relationships."

(ii) Why do the authors use the term 'corticokinematic coherence', although they use the EMG as a reference? I was expecting a kinematic recording from the title such as an accelerometer. Would corticomuscular coherence not be more intuitive here? Moreover, do we really know how much of CKC is really proprioception and how much is motor command? And how far can we disentangle both?

Response to (ii): We agree with the Reviewer that referring to CKC when relying on (rectified) EMG signals is somewhat confusing. However, previous work has demonstrated that a similar coupling is uncovered with acceleration, force, pressure, and EMG signals (Piitulainen et al. (2013). Coherence between magnetoencephalography and hand-action-related acceleration, force, pressure, and electromyogram). Importantly CKC and corticomuscular coherence (CMC) differ in two main aspects: CKC is observed during repetitive movements and CMC during sustained isometric contractions, and CKC peaks at movement frequency (~1 to 4 Hz), while CMC tends to peak between ~15 and 40 Hz. Hence, we believe that CKC is the adequate term to describe our results. We have added some information to the revised manuscript to convey these ideas.

See Introduction (lines 54-57):

"A promising method in that respect is corticokinematic coherence (CKC), the coupling between cortical activity and limb kinetics or kinematics that occurs during repetitive movement tasks at movement frequencies (Bourguignon et al., 2011, 2012; Piitulainen et al., 2013a)."

(lines 80-81):

"In this study, we estimated CKC between cortical activity and rectified EMG signals, as done previously (Piitulainen et al., 2013b; Bourguignon et al., 2019) [...]"

See Methods (lines 221-223):

"Noteworthily, a rectified EMG signal picks up movement rhythmicity much like an accelerometer, so that its coherence with EEG signals at movement frequencies can be safely termed CKC (Piitulainen et al., 2013b; Bourguignon et al., 2019)."

We also provide more background information on the studies suggesting that CKC hinges on proprioception (see lines 57-58):

"Indeed, studies comparing CKC during active and passive movements and using directionality approaches suggest that CKC specifically assesses the cortical processing of somatosensory proprioceptive afferences (Piitulainen et al., 2013a, 2018a; Bourguignon et al., 2015)."

(iii) Already in the abstract the authors might want to report statistical results for their correlation analyses. This would be useful for the readers.

Response to (iii): We appreciate this helpful comment and have incorporated the relevant statistics in the abstract.

(iv) The term 'motricity' in the discussion might not be familiar to all readers and might benefit from some explanation.

Response to (iv): 'Motricity' is now replaced by 'motor control', which we had used everywhere else in the text. It will hopefully be easier to comprehend for readers.

(v) I'm not sure that faithful/faithfully are the most fitting words as used here.

Response to (v): We thank the reviewer for ensuring clarity in this manuscript. The first instance of faith/faithfully was deemed not necessary and deleted. The second instance was changed to 'robust', which more clearly articulates our interpretation: "The relationship between CKC and task performance might also be explained by a more robust cortical representation of proprioceptive afferences in the best performers." (line 412)

(vi) Please use 's' for seconds throughout the entire manuscript including legends instead of spelling it out.

Response to (vi): All instances of seconds have been corrected to 's.'

(vii) The citations Bernshtein (1967) and Hyvärinen et al (2001) are incomplete.

Response to (vii): The citations are now complete - we thank the reviewer for pointing out these reference mistakes.

-

Reviewer #2:

In this study, the authors carried out EMG and EEG recordings in human subjects performing a gross motor task ("BBT"), and separately had the same subjects perform a fine motor task ("PPT"). In addition to the motor performance scores themselves, they carry out a number of rigorous quantifications based on the EEG and EMG data acquired during the BBT task. These include: (i) muscle coordination - a measure of how well the activation pattern of each muscle predicted that of others, (ii) regularity - a measure of the periodicity of each muscle activation, and (iii) "CKC" - the coherence between the EMG and EEG data as a function of frequency. Their main finding is that high CKC values at the first harmonic of the movement frequency in the primary sensorimotor area was associated with better performance in the BBT task (Figure 4 and Figure 5A), which is an interesting result and should be reported.

However, the data for the PPT task and its relationship with the rest of the data are, in my opinion, much harder to interpret since they represent an independently acquired dataset (albeit from the same subjects). The authors find that CKC values have no relationship with PPT performance (Figure 5B). I am not sure why we should expect one in the first place, since the PPT is an entirely different task and BBT and PPT scores were only weakly correlated with each other. The authors also find that subjects with high PPT scores tended to have lower CKC values than one would expect given their BBT scores - but this is also difficult for me to interpret since I lack a clear intuition for how the measured CKC values might relate (if at all) to the PPT task.

Response to general comment: We thank the reviewer for this comment. Using the framework of a previous study in which CKC values as measured during passive ankle movement were compared to postural stability (Piitulainen et al. (2018). Cortical proprioceptive processing is altered by aging. Frontiers in aging neuroscience), we find it reasonable to compare CKC to PPT performance. In the above study, even though CKC was taken from a passive-foot movement task, the corresponding CKC values were significantly related to postural sway. This work suggests that CKC measurement represents a global index of cortical proprioceptive processing, whereby we may possess some inherent cortical processing ability that can be measured in one task and show significant relationships with other features of motor control. Accordingly, it is possible that CKC is not related to PPT performance because of the features specifically contributing to the task itself. Alternatively, we agree that we cannot rule out the possibility that CKC was more strongly associated with BBT compared with PPT because it was measured during the BBT. In summary, we still believe there is some merit to including correlations with PPT, and that more discussion should have been included on the significant fact that CKC was measured in-task for BBT and off-task for PPT. The revised manuscript incorporates such discussion (see lines: 500-510)

"Lastly, it could be argued that CKC should be interpreted only in the context of the task from which it is derived - BBT in our case. Nevertheless, previous work has established that CKC measured in one context could be related to performance in another completely different task. Specifically, CKC measured during passive ankle movements was found to be related to the amplitude of standing postural sway (Piitulainen et al., 2018b). Hence, CKC may represent a global index of proprioceptive processing, suggesting it is warranted to compare CKC across task types. Therefore, in aiming to develop a nuanced understanding of CKC's relationship to gross and fine motor control, we compared CKC magnitude to BBT and PPT performance. Future work investigating how CKC varies across tasks in the same individual should clarify to which extent CKC reflects some innate cortical proprioceptive processing ability as opposed to task specific demands."

Minor:

In the Results section, the authors report that several measures were correlated with each other and that this was somehow corrected for (for instance, correlation between coordination measure and EMG modulation depth). It is unclear to me how this correction was carried out. It might also be helpful to directly show the result of the correction by plotting the correlated measures against each before and after the corrections.

Response to minor comment: We agree that clarifications were needed with regards to the correction approach. These are now added in the Methods of the revised manuscript (see lines: 265-271):

"Potential confounding factors for regularity, coordination, and CKC (our dependent variables) were assessed by correlation and corrected for when the correlation was statistically significant. Specifically, when a significant correlation was identified with a confounding variable, the mean subtracted confounding variable was regressed out of the dependent variable, resulting in a new corrected dependent variable. When more than one confounding factor were identified, they were regressed out sequentially, taking care of orthogonalizing each of them with respect to the previously removed ones."

In addition, we have amended Figure 5, and included plots of the EEG-SNR corrected CKC and the motor task scores in accordance to the Reviewer's comment, now directly showing the result of the correction.
